# Academia Europaea Position Paper on Translational Medicine: The Cycle Model for Translating Scientific Results into Community Benefits

**DOI:** 10.3390/jcm9051532

**Published:** 2020-05-19

**Authors:** Péter Hegyi, Ole H. Petersen, Stephen Holgate, Bálint Erőss, András Garami, Zsolt Szakács, Dalma Dobszai, Márta Balaskó, Lajos Kemény, Shuang Peng, Joao Monteiro, András Varró, Tara Lamont, Jeffrey Laurence, Zoe Gray, Andrew Pickles, Garret A. FitzGerald, Christopher E.M. Griffiths, Jacek Jassem, Dmitri A. Rusakov, Alexei Verkhratsky, Andrea Szentesi

**Affiliations:** 1 Institute for Translational Medicine, Medical School, University of Pécs, 7624 Pécs, Hungary; eross.balint@pte.hu (B.E.); andras.garami@aok.pte.hu (A.G.); szaki92@gmail.com (Z.S.); dobszai.dalma@gmail.com (D.D.); mmbalasko@gmail.com (M.B.); szentesiai@gmail.com (A.S.); 2 School of Biosciences, Cardiff University, Cardiff CF10 3AX, Wales, UK; PetersenOH@cardiff.ac.uk; 3 Clinical and Experimental Sciences, Faculty of Medicine, University of Southampton, Southampton SO16 6YD, UK; S.Holgate@soton.ac.uk; 4 Department of Dermatology and Allergology, University of Szeged, 6720 Szeged, Hungary; kemeny.lajos@med.u-szeged.hu; 5 Department of Pathophysiology, School of Medicine, Jinan University, Guangzhou 510632, China; pengshuang@jnu.edu.cn; 6 Nature Medicine, New York, NY 10004-1562, USA; joao.monteiro@us.nature.com; 7 Department of Pharmacology and Pharmacotherapy, University of Szeged, 6720 Szeged, Hungary; varro.andras@med.u-szeged.hu; 8 NIHR Dissemination Centre, University of Southampton, Southampton SO16 7NS, UK; tara.lamont@nihr.ac.uk; 9 Weill Cornell Medical College, New York, NY 10065, USA; jlaurenc@med.cornell.edu; 10 National Institute for Health Research, Southampton SO16 7NS, UK; zoe.gray@nihr.ac.uk; 11 Biostatistics and Health Informatics, King’s College London, London WC2R 2LS, UK; andrew.pickles@kcl.ac.uk; 12 Institute for Translational Medicine and Therapeutics, Perelman School of Medicine, University of Pennsylvania, Philadelphia, PA 19104, USA; garret@upenn.edu; 13 Dermatology Centre, NIHR Manchester Biomedical Research Centre, University of Manchester, Manchester M13 9WU, UK; christopher.griffiths@manchester.ac.uk; 14 Medical University of Gdańsk, 80-210 Gdańsk, Poland; jjassem@gumed.edu.pl; 15 UCL Queen Square Institute of Neurology, University College London, London WC1N 3BG, UK; d.rusakov@ucl.ac.uk; 16 Faculty of Biology, Medicine and Health, University of Manchester, Manchester M13 9PT, UK; Alexej.Verkhratsky@manchester.ac.uk; 17 Achucarro Centre for Neuroscience, IKERBASQUE, Basque Foundation for Science, E-48940 Bilbao, Spain

**Keywords:** translational medicine, translation, healthcare, science, knowledge, communication, interdisciplinary

## Abstract

Introduction: Translational science has gained prominence in medicine, but there is still much work to be done before scientific results are used optimally and incorporated into everyday health practice. As the main focus is still on generating new scientific data with financial resources primarily available for that purpose, other activities that are necessary in the transition from research to community benefit are considered less needy. The European Statistical Office of the European Commission has recently reported that 1.7 million people under 75 years of age died in Europe in 2016, with around 1.2 million of those deaths being avoidable through effective primary prevention and public health intervention. Therefore, Academia Europaea, one of the five Pan-European networks that form SAPEA (Science Advice for Policy by European Academies), a key element of the European Commission’s Scientific Advice Mechanism (SAM), has launched a project to develop a model to facilitate and accelerate the utilisation of scientific knowledge for public and community benefit. Methods: During the process, leaders in the field, including prominent basic and clinical researchers, editors-in-chief of high-impact journals publishing translational research articles, translational medicine (TM) centre leaders, media representatives, academics and university leaders, developed the TM cycle, a new model that we believe could significantly advance the development of TM. Results: This model focuses equally on the acquisition of new scientific results healthcare, understandable and digestible summation of results, and their communication to all participants. We have also renewed the definition in TM, identified challenges and recommended solutions. Conclusion: The authors, including senior officers of Academia Europaea, produced this document to serve as a basis for revising thinking on TM with the end result of enabling more efficient and cost-effective healthcare.

## 1. Introduction

### 1.1. Utilisation of Science

Scientific thinking and the utilisation of scientific results have become indispensable in almost all areas of life. There are hardly any areas of our daily activity where decisions informed by scientific evidence are not better and more economically viable, even in the short term, than those based on individual experience. Currently, no trustworthy decisions can be made without the use of evidence to support them. In order to incorporate evidence into daily practice, scientific knowledge must be translated into a language which is understandable for decision makers and the general public. 

While 50 years ago, in 1969, 215,347 articles were listed in PubMed, this number increased to 1,400,975 in the year of 2019, and undoubtedly will continue to grow. Safe integration of scientific results into everyday healthcare necessitates a new way of thinking. 

### 1.2. The Evolution of Medicine

**Figure 1:**
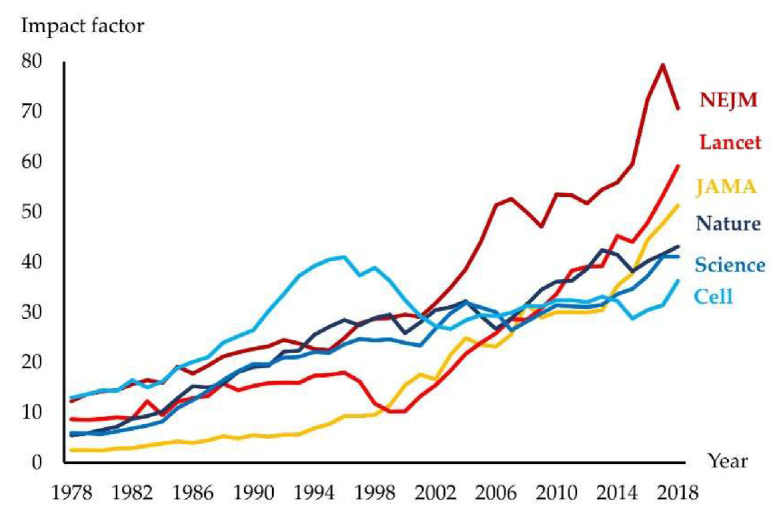
The impact factor for leading international journals. JAMA: Journal of the American Medical Association; NEJM: The New England Journal of Medicine

One of the greatest challenges of the last century has been to understand how the human body works at the organ, cellular and subcellular levels (anatomy, physiology, biochemistry, biophysics, etc.) and then to investigate the development of, and the mechanisms underlying, different diseases (pathology, pathophysiology, etc.). Without this knowledge, clinical science is unable to evolve. Most people held the view that “real science” takes place at the bench, and not at the bedside. In the 20th century, the impact of journals publishing basic discoveries was generally higher than for those addressing clinical and health sciences [1]. In the 21st century, this trend has reversed. Indeed, an increasing number of biomedical journals have a clinical orientation, and along with this, transferring scientific knowledge into clinical practice has become one of the most important challenges in medicine [2]. This view has resulted in much greater funding of clinical compared to basic sciences [3]. Furthermore, clinical scientists typically produce many more papers than basic scientists [4]. This inevitably influences numbers of citations reflected in the development of impact factors (IF) for basic- and clinically-oriented journals (Figure 1). 

Importantly, the high IF of many clinical journals does not necessarily indicate higher quality of clinical compared to basic science, because citation numbers in a particular area inevitably also reflect the size of that field. The last assessment of research quality in the UK (REF2014) performed by a distinguished international panel demonstrated that the proportion of top-quality research outputs in the biological sciences was higher than in clinical medicine [3].

### 1.3. The Evolution of Translational Medicine

These bidirectional changes in basic and clinical sciences have been accompanied by increasing attempts to evaluate the applications and usefulness of discovery science rather than their originality. Both grant committees and politicians have encouraged scientific activities that could be translated into practical applications. Therefore, in some jurisdictions, the possibility of obtaining, e.g., a purely basic physiology grant has dramatically decreased as compared to that with a potential clinical value. To satisfy this public demand, a new category of science, translational medicine (TM) emerged in the 1990s and has subsequently developed tremendously. The fashion for translation in medicine is also illustrated by the fact that currently 32 journals in their title include the word ‘translational’. However, many clinicians are concerned that most translational discoveries may remain hypothetical if they are not translated into clinical trials [5]. In June 2000, a roundtable discussion addressed the issue of different TM definitions [6]. Due to the rapid evolution of TM within the past two decades, these definitions have been subjected to ongoing revisions. On many occasions, TM has also been conflated with translational science or research. Hitherto, three main concepts have been formulated [7].

#### 1.3.1. One-Way Concept 

In this, TM is considered as a “bench-to-bedside” enterprise, in which basic discoveries can lead to development of new drugs, devices and treatments for patients [5,8]. This concept has been further developed by clinicians who call TM a tool, in which research can be translated into clinical practice [9]. 

#### 1.3.2. Two-Way Concept

The “bench-to-bedside-to-bench” concept also incorporates the use of feedback, or clinical pull, from the bedside as a basis for further fundamental research [10,11,12]. In this concept, basic researchers have highlighted the importance of clinical knowledge and unmet needs in developing basic research projects.

#### 1.3.3. One-Way Multiple Steps Concept 

The research community has realised that translating molecular sciences to benefit patients cannot be achieved in one step. Five phases (T0–4) have been created: T0: basic biomedical research, T1: translation to humans, T2: translation to patients, T3: translation to practice and T4: translation to communities. This transitional concept has highlighted the importance of community more extensively, including patients, healthy populations, participants in the healthcare system and public bodies [13,14].

Notably, none of the theories cover the whole TM cycle which starts from a clinical question from the public or the bedside, and finishes with an applicable answer, which can be used to improve the health of the community. Furthermore, there are still numerous missing steps in the cycle, which dramatically slow down the transition of new scientific knowledge into community benefit. For example, (1) there is little communication between participants of TM, such as physicians, basic scientists, pharmaceutical companies, clinical scientists, economic and political decision-makers and communities [15]. (2) Existing models also presume distinct stages and a linear approach, whereas the reality of translational medicine is more iterative with multiple feedback loops in a dynamic interactive and complex system. (3) Some of the areas are over-represented and possibly over-supported, whereas others receive insufficient funding and opportunities to develop, thus further hampering the transition process in response to the health burden of specific diseases and co-morbidities. (4) Almost everyone wants to promote new science, but there is hardly any finance available for promoting the uptake and utilisation of scientific results. (5) Academies and universities handle the relative importance of publications from the different phases of research in uneven and sometimes contradictory ways, usually with little reward for translating scientific results into everyday practice [16]. (6) There remain unresolved conflicts and ignorance surrounding unrestricted access to research outputs such as journal articles. Although protection of intellectual property and commercialisation is necessary for many discoveries to be translated, they do not favour quick transition. (7) Interdisciplinary units consisting of professionals in medicine, IT, biostatistics, health economics, and data management and analyses, governance and ethics, are unevenly established in medical schools, hospitals and TM centres [17]. Finally, (8) there is a scarcity of complex educational materials in TM. Indeed, physicians and other healthcare professionals are unable to interpret the usability and limitations of scientific knowledge/evidence without an understanding of the underpinning scientific methodologies used [18]. 

Importantly, medicine should be open to criticism and challenge. One important issue is the so-called reproducibility crisis. Several cases have contributed to the difficulties of translating basic science results into improvements in clinical care. This problem has been particularly pronounced in cancer-related signal transduction studies [19], mostly on cell lines, and it deserves serious attention here and in other cases as well [20]. For example, samples in TM studies cannot be compared with each other without limitations due to the different pre-analytical treatments of the sample.

In November 2018, Academia Europaea decided to review the field of science utilisation in Medicine in order to make recommendations to participants (countries, politicians, insurance companies, medical practitioners and other health care workers) to accelerate the use of scientific results for health benefits and make our healthcare systems more effective and cost-effective. Here, we present a proposal for comprehensive and usable TM model and cycle that could be valuable for all TM participants. We emphasise that our healthcare systems can only be improved if all areas of TM receive the appropriate attention and support.

## 2. Methods

### 2.1. Initiation and Work Packages

The project was launched by the Centre for Translational Medicine led by Péter Hegyi and discussed and modified by Ole H. Petersen and Stephen Holgate. The framework that served as a basis for the project was accepted by the Academia Europaea Board in Barcelona in November 2018. Six work packages were developed: WP1: Healthcare: different levels of translational healthcare institutions were determined according to the level of involvement in translational research; WP2: Science: summarised the methodologies leading to (i) discoveries of new mechanisms and targets, (ii) development of new drugs and interventions and (iii) information concerning the safety and effectiveness of interventions; WP3: Knowledge: summarised the different categories of knowledge publications, including (i) discussions, commentaries and individual opinions; (ii) reviews; (iii) systematic reviews; (iv) textbooks: publications of specialist working groups; (v) EBM Guidelines; and (vi) national and local protocols; WP4: Communication: defines the target audiences, channels of communication and recommends measures for improvement; WP5: Interdisciplinary: summarised the categories of essential fields for successful research support; and WP6: Academy: developed recommendations for universities and academies concerning the usability of different scientific methods (i.e., systematic reviews, meta-analyses, and case reports).

Members of the Academia Europaea’s Life Sciences class were invited to participate. Invitations were also sent to journals related to TM. Members who accepted the invitation were also allowed to nominate additional experts. Altogether, 22 experts (authors) accepted the invitation. 

### 2.2. Chronological Order of the Work

The following schedule was followed: Phase 1: February–April 2019; identifying specific questions, challenges and unmet needs in TM. WPs developed draft summaries and recommendations for their respective fields, which were then discussed within the WPs via e-mail. Phase 2: May–June 2019; The preparatory phase of the project ended with a video conference discussing WP issues. Phase 3: July–August 2019; drafting the results of the video discussions and returning them to members. Phase 4: 22–23 September 2019; holding an open conference in Budapest in the premises of the Hungarian Academy of Sciences, with all members invited to join either locally or via Internet. Phase 5: October–December 2019; drafting the article. Phase 6: February 2020; modifying and agreeing on the article. Each WPs was discussed until full consensus was achieved.

## 3. Results 

### 3.1. The TM Cycle

**Figure 2:**
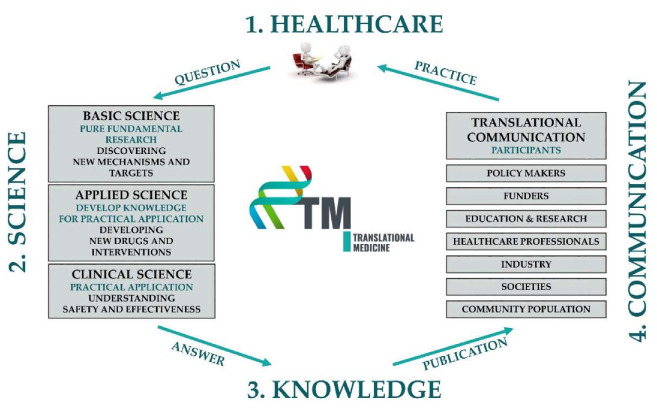
The translational medicine (TM) cycle.

The TM cycle contains four important elements: TM Healthcare, TM Science, TM Knowledge and TM Communication (Figure 2). TM Healthcare is the starting point where clinical questions arise. Questions can only be answered through scientific activities in which researchers use tried and tested basic, applied or clinical scientific methodologies in an objective and reproducible way. The results thus generated undergo a rigorous review process to ensure quality and validity and are published in a written form to inform the scientific community. However, they clearly need to be summarised in a digestible way for all healthcare stakeholders, including physicians, nurses, patients, healthy individuals and decision-makers. The deliverables of this process should include evidence-based guidelines, systematic reviews and evidence-based patients’ information leaflets. These publications should also undergo a scholarly peer-reviewed process. They need to be communicated and disseminated to the target audiences in their own native languages and in an understandable format. Finally, healthcare participants need to use the information in practice to achieve potential benefits. Importantly, if any element of this cycle is missing, no healthcare improvement can be accomplished (Table S1).

#### 3.1.1. TM Healthcare

Healthcare is the starting and finishing point in TM. Ample evidence points to clinical research resulting in better outcomes and patient experience in healthcare [21,22,23]. Currently, only a narrow area of patient care is involved in scientific activities or clinical research. The widespread adoption of science in patient care is essential for improving the quality and cost-effectiveness of healthcare. 

##### Definition of TM Healthcare

TM Healthcare should be undertaken in a purpose-designed and structured setting that is able to permeate through the whole healthcare system. It can significantly boost the efficiency of clinical research and promote efficient communication and cooperation between all stakeholders, including clinical and basic scientists, patients, government agencies, and insurance and pharmaceutical companies. It should also aim to involve patients in a bidirectional discussion and communication with all other stakeholders. TM Healthcare aims to improve patient care and clinical research; this means high-quality patient care, driven by and contributing to high-quality clinical research. 

##### Multidisciplinary Care

However, the success noted above can only be achieved via multidisciplinary agreement, when all healthcare providers agree on evidence (science)-based patient management. A challenging example is the rapid evolution of cancer care where domain expertise in radiotherapy, chemotherapy, immunotherapy and surgery need to be routinely integrated and sequenced with the potential for infectious, haemostatic and GI complications. A specific example of multidisciplinary collaboration is the case of a pregnant woman suffering from acute pancreatitis in the summer of 2018 in one of the newly established TM Healthcare centres in Hungary (Szent György Teaching Hospital of Fejér County, Székesfehérvár). She presented with severe abdominal pain. An examination showed that the foetus was healthy in the 39th week of pregnancy. Abdominal ultrasound (US) and laboratory studies showed gallstones and features of acute biliary pancreatitis, but no inflammation of the biliary system. She gave birth to a healthy new-born baby after a quick recovery from mild pancreatitis. Biliary ductal stones were ruled out by magnetic resonance cholangiopancreatography, and she underwent a cholecystectomy a few days later. Within five days, the happy, healthy mother and new-born child went home and neither of them required further hospitalisation. Without the urgent, coordinated and expert cooperation of the emergency physician, internist, gastroenterologist, obstetrician–gynaecologist, radiologist and surgeon, this could not have happened. Notably, it is rare that expertise spills over from one domain into the other and yet the real challenge is how to combine these approaches most safely to achieve efficiency and added value. 

Another form of multidisciplinarity could be when scientists and medical personnel work together for the implementation of scientific results in patient care. The centres having such multidisciplinary teams should be evaluated and good centres should be differentiated also by financial means. Examples are mentioned in Section 3.2.

##### Patient Care in Specialised, High Volume Centres (Tertiary Care)

This case also illustrates that large healthcare centres are particularly well suited to be the leading TM Healthcare institutions. One of the main conclusions of our project is that we need to bring scientific thinking to the hospital level, as it will help to assess the efficiency and quality of treatment for a given diagnosis. Usually, tertiary centres where a greater number of patients are treated, provide higher quality patient care [24,25]; thus, in countries where TM is not yet implemented at the hospital level, tertiary centres may be ideal locations to start implementing the TM Healthcare system.

##### Involvement in Healthcare Data Collection 

Continuous data recording and data monitoring is extremely important in patient care. One of the great benefits of data recording is that it can be used for quality monitoring, which itself significantly improves the quality of patient care, both in terms of disease outcomes and patient experience. Another important task of data capture is the planned collection of data from patients in registries and clinical trials for future analyses.

A special aspect of TM Healthcare facilities is that they use an advanced data acquisition infrastructure and employ highly qualified data administrators, programmers and analysts. Data are collected by a data management team under the guidance of a TM researcher. They use standardised and structured data recording forms and a computerised system. Particular attention must be paid not only to the quantity, but also to the quality of the data processing pipeline, since incorrect data can give a false sense of reality and lead to incorrect conclusions. TM Healthcare facilities place a special emphasis on the quality of data collection. 

##### Involvement in Biological Sample Collections 

Biological samples collected from individuals are essential components of basic, applied and clinical research. It is thus important that TM Healthcare facilities have the appropriate infrastructure and trained staff to collect the various biological samples. Additionally, biological samples should be collected according to international guidelines and standards of the International Organization for Standardization. The TM Healthcare centres should closely cooperate with research centres to promote scientific activities which was illustrated nicely by recent research activities in circadian medicine [26]. 

##### Financing TM Healthcare

TM Healthcare needs to be funded from diverse sources. Activities that are part of general patient care and are already summarised in the evidence-based guidelines should be financed by national and private health insurance. Activities intended to achieve new scientific results should be funded by institutional, national and international grants and funds. Importantly, a special emphasis should be put on raising budgets for science in Eastern and Central European countries. This provides additional resources for staffing, consumables and facility development. It is important that, while adhering to state-of-the-art evidence-based guidelines, the cost-effectiveness of patient care improves, resulting in a release of resources.

##### Levels of Progressivity in TM Healthcare

We believe that overall patient care would be better and more cost-effective if all healthcare facilities worked at the same high level as TM Healthcare centres. However, we are aware that this can only be done step by step, since it initially requires additional resources to develop the system, and participants need to have a chance to grow accustomed to new science-based decision-making and scientific thinking. Therefore, we consider it necessary to differentiate between the levels of TM Healthcare, which may help (1) to stimulate the transition of scientific results to community benefits, (2) to assess the efficiency of centres and to formulate expectations from healthcare providers and (3) to help governments, universities and grant committees to set up specific development plans.

Basic-Level TM Healthcare Institution

The healthcare institution employs at least one full-time clinical research administrator and provides data for registries and clinical trials. They use evidence-based medicine (EBM) guidelines in healthcare in a multidisciplinary and timely manner. 

Intermediate-Level TM Healthcare Institution

The healthcare institution employs at least one full-time clinical research administrator and a clinical researcher (designated clinical researcher, Ph.D. student or designated physician). These centres collect clinical data and biological research samples for scientific activities. As pointed out earlier, they must use the EBM guidelines in healthcare in a multidisciplinary and timely manner.

Advanced-Level TM Healthcare Institution

Besides the criteria written in the intermediate-level TM Healthcare institution, advanced-level institutions not only participate in research activities, but the teams working here plan, launch and maintain disease registries and clinical trials. An interdisciplinary working group (see III.2.1) must also be formed.

##### Benefits of TM Healthcare

There are many suggestions and recommendations on how research impact or value can be enhanced in existing healthcare systems [27,28]. TM Healthcare can increase the relevance of collaborative clinical and basic research projects and can significantly reduce the time required to answer a clinical question arising at the bedside. It can maximise the level of evidence provided by medical research. As a result, improved prevention, diagnosis and treatment make patient care safer and better. TM providers also ensure that clinical data is collected in a structured manner with high-quality standards. Given that TM Healthcare also acts as a bridge between science and patient care, scientific results are incorporated into day-to-day patient care in a significantly shorter time than in a general healthcare system, making it more efficient and cost-effective. However, the biggest winners of TM Healthcare are always the patients and healthy populations.

#### 3.1.2. TM Science

Many discoveries in basic research have not progressed adequately to influence patient care, while clinically relevant questions have not been addressed through the arsenal of basic research methodologies. Moreover, scientifically validated drugs and interventions have not been properly transmitted to clinical practice to benefit public health. Regional differences have also occurred in the dominance of certain areas of science (e.g., in Western Europe basic science funding is inadequate in relation to the scientific opportunities that exist, whereas clinical science is less developed in Eastern Europe). These gaps need to be filled. 

##### Definition of TM Science 

TM science should be defined as any activity that generates new discoveries or observations, which help to form our knowledge of the human body and its interactions with the environment. It should also carry a clear hope of attaining novel achievements for the benefit of human health. TM science must aim for principles, such as objectivity and reproducibility. 

According to the most recent definition set down by the National Centre for Advancing Translational Sciences (NCATS) at the National Institutes of Health, translation in science is “the process of turning observations in the laboratory, clinic and community into interventions that improve the health of individuals and the public” [29]. Translational science is a discovery-driven research field (i.e., discovery science), in contrast to purely observational (i.e., not hypothesis-driven) sciences, which do not aim to solve a particular problem. The border between translational science and other sciences is, however, not always obvious. At the time when an observational discovery is made in pure basic research, it can be difficult to predict whether it will have an impact on human health. Therefore, if one takes a wider view, a use is ultimately sought for all discoveries, so that they can be translated for the improvement of human health (whether we know their use in advance or not). 

**Figure 3:**
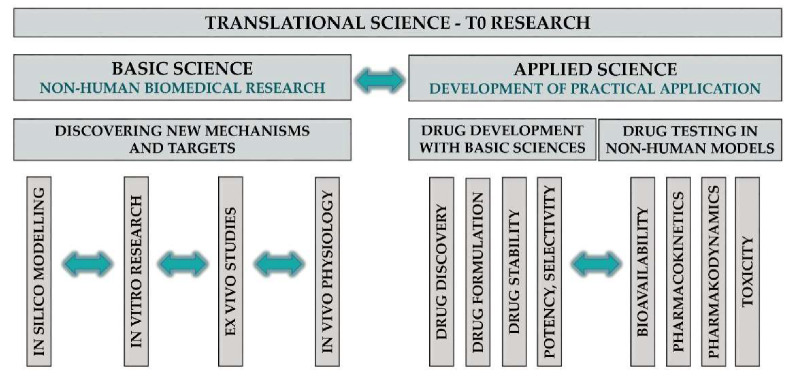
Translational science T0 research.

Translation can be achieved by a plethora of different scientific research approaches at different levels of the process; these are commonly referred to as translational research. Waldman and Terzic [30] defined the phases of the TM continuum, which have been accepted and used by many organisations, including the NCATS. T0 research includes basic and applied sciences (Figure 3).

To establish and understand the different categories in TM is not only important from a scientific point of view, but also (and perhaps to a greater extent) for decision-makers, who are responsible for distributing funds and resources to these categories.

Basic Science in TM 

Basic science in TM is the discovery of novel mechanisms and targets in clinically relevant areas with any basic biomedical research tools. Basic research tools range from in silico through in vitro to in vivo techniques. These include, but are not restricted to, mathematical modelling, molecular biology (e.g., gene sequencing, RNA and protein expression, immunohistochemistry, signal transduction and enzyme activation/inhibition), cell physiology (e.g., calcium imaging, patch clamp and organoid research) and in vivo animal experiments (e.g., integrative physiology, neurophysiology, behavioural tests and experimental models of diseases). 

Applied Science in TM 

**Figure 4:**
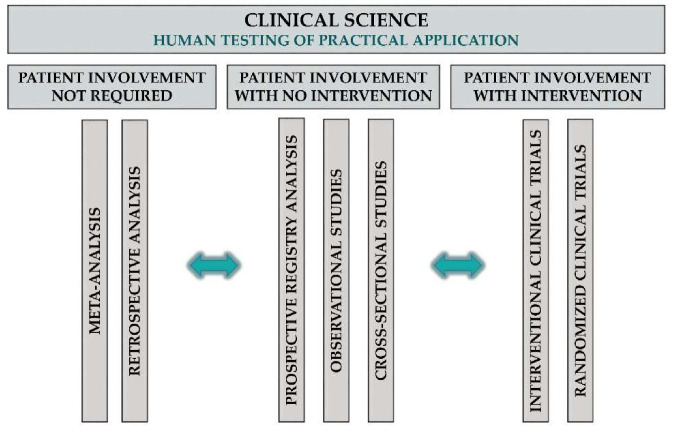
Clinical science in TM.

Applied science in TM entails the development of new drugs or interventions, which can be divided into two main categories: (1) non-clinical drug development, including drug discovery (e.g., synthesis of highly specific and potent compounds for a target), formulation (e.g., solubility and coating) and stability (e.g., drug shelf time) studies; and (2) preclinical testing of developed compounds, mainly in animal models usually conducted in rats or mice, then also in non-rodent mammals (e.g., dogs) and primates. Bioavailability, pharmacokinetic and pharmacodynamic, as well as toxicity studies, are included in this phase. T1 and T2 research involves translation to humans and to patients, respectively; both types are clinical studies (Figure 4). 

Clinical Science in TM 

Clinical science in TM can mean any scientific research that uses data from human subjects to test and further develop the output of applied science in humans, including, but not restricted to, retrospective and meta-analysis of previously collected data, prospective registry studies, observational and interventional studies and phase I to III clinical trials. 

The T3 and T4 steps in translational research aim at translating discoveries into clinical practice and to communities, respectively. These steps, also involving an envisioned T5 step, which goes beyond the public health model of care and extends to the social health model [31], are also essential in the translation process. Many of the greatest innovations come in the way of how we deliver services instead of discovering new molecules or interventions. For example, patient education plays a crucial role in the prevention of recurrence in pancreatitis, and early diagnosis of cancer is extremely important for prognosis. The later (T3–T5) steps in TM aim at better and more efficient dissemination of discoveries, which can also result in system-level changes in healthcare, as well as in public health. 

Importantly, whereas in basic research new data are generated and conclusions mostly drawn from the analysis of newly generated data, in clinical science researchers often use existing data (e.g., data in publications, in patient registries and in national health systems) in their analysis. The latter research activities can also generate new information and new discoveries; therefore, all publications describing new, previously unknown results should be considered as original publications, regardless of whether these analyses are based on newly generated or already existing data. Analysing large datasets is a powerful mechanism for refining and prioritising the infinite number of hypotheses that can be addressed in a randomised controlled trial [32,33]. 

In many cases, basic research is often translated first to applied science before the results are implemented in patient care. In such situation the real work where the implementation started, hardly receives any citations. Rewarding applied sciences and implementation of basic research findings with higher impact factors could also help the translation process.

#### 3.1.3. TM Knowledge 

The recent explosion in the number of scientific publications has raised a new issue: while thousands of scientific articles are published daily, readers cannot keep pace with their sheer number, not even in their own areas of interest [34]. The content of papers without a readership remains unutilised and will have no influence on further research or on clinical practice [35].

This phenomenon has provided the basis for summary publications, which have become popular and highly cited [36]. In fact, it has long been known that the IF of journals, as well as the citation record of individual scientists, are more impressive when review articles dominate [37,38,39]. Unfortunately, many review articles contain inaccuracies, are biased in their selection of papers cited and do not refer to the original sources of the discoveries that are discussed, but often refer to other review articles [40]. This has led to the proposal for the generation of Evidence Reviews with precise referencing to the original peer-reviewed literature [40]. Although review articles are more frequently cited than original papers, they are generally not nearly as carefully assessed as original articles.

In this paper, we attempt to define a new class of scientific publications, TM Knowledge. These publications summarise and qualitatively synthesise all current scientific information on a well-defined topic to advance the translation of findings into research and practice. They also aim to deliver relevant information in an easily available and digestible, well-organised format for scientists of any discipline and lay readers, such as politicians and patients.

For healthcare-related disciplines, TM Knowledge publications should use the basic principles of evidence-based medicine (EBM), including reproducibility and transparency in methodology. As mentioned above, summary (review) papers are often deficient; however, if executed and peer-reviewed with care, a review paper can be as valuable as an original research paper with primary data. However, we should be aware that journals may inflate their metrics by publishing a large number of reviews [41].

##### Definition of TM Knowledge 

TM Knowledge aims to summarise the discoveries of TM Science to facilitate the translation of scientific findings to community benefits. Its language and the way of writing should be chosen in a way that is understandable to the target audience. 

Opinion leaders might facilitate the transition. Importantly, the publication explosion accelerates the turnover of scientific information: if not kept up-to-date (with a ‘living’ summary), summary publications are no longer capable of fulfilling their role. Therefore, if new findings emerge, they should be integrated into publications as soon as possible to allow the translation of modified conclusions. The exploding use of open access preprint repositories for the biological sciences such as bioRxiv can also help to shorten the time from discoveries to their utilisation. However, since these pre-publications are not peer-reviewed, they should be handled with caution [42]. 

**Figure 5:**
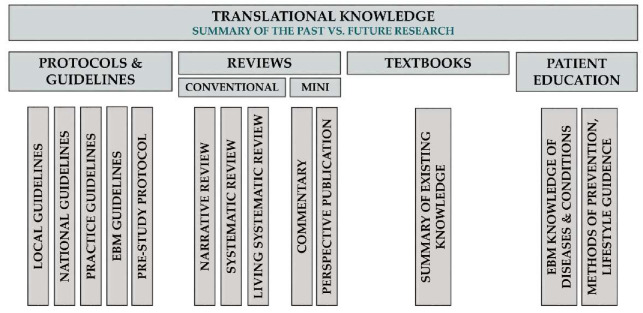
Translational knowledge.

To treat different scientific genres properly, we propose the following classification of TM Knowledge publications (Figure 5).

##### Classification of TM Knowledge Articles

Review Articles 

The aim of review articles is to compile evidence from the scientific literature about a well-defined problem, question or topic. Conventional reviews can draw conclusions from current evidence, should rate the quality of evidence and should discuss the implications of findings for research and practice, but should not formulate and grade the level of recommendations, as do guidelines. Systematic reviews form a subtype of reviews with the aim of summarising all available evidence collected through a systematic literature search in a standard, reproducible way, as proposed by the Cochrane Collaboration. Living systematic reviews are continually updated, incorporating relevant new evidence as it becomes available, whereas non-systematic narrative reviews share the purpose of systematic review articles, but they do not adhere to the standard, reproducible methodology of systematic reviews (main target audience: guideline developers, basic and clinical scientists, economic decision-makers and industry). It should be emphasised that meta-analyses that are already mentioned in the clinical science category can be important components of a systematic review procedure. However, since a meta-analysis contains a research question, a hypothesis, rigorous mathematical methods and a results section, and often ends up with new discoveries, it should be counted as a scientific rather than a knowledge article.

Correspondence Publications 

Their aim is to react to published material by either confirming or refuting it. If a commentary contains original data generated by the authors, the publication falls under TM Science; otherwise, it is part of TM Knowledge and should be considered as a mini-review (main target audience: authors of the commented articles, the readership of the journal and editors).

Hypothesis Publications 

These are mini-reviews aiming to provide a narrative summary that inspires further research. 

Pre-study Protocols 

The aim of pre-study protocols is not only to describe clinical trial design and protocol, but also to provide a summary of the literature with a proper justification for the trial. Ideally, each clinical trial should be registered and preceded by a systematic literature search, the results of which should be summarised in the pre-study protocol (main target audience: editors, reviewers and other researchers contemplating studies in the same area or with similar design).

Practice Guidelines 

Practice guidelines provide a step-by-step guide on what to do in specific clinical settings or in the management of well-defined conditions. Guidelines can be tailored to local and national levels (main target audience: clinical practitioners). EBM guidelines are mainstays of evidence-based medicine. These guidelines share the roles of practice guidelines but must adhere to reproducible standards (e.g., the Grading of Recommendations, Assessment, Development and Evaluations (GRADE) system) when rating quality of evidence and formulating the strength of recommendations (main target audience: clinical practitioners and scientists) [43,44]. Unfortunately, there is a huge gap between the publication of a guideline and its actual implementation [45]. Furthermore, guideline adherence is poor in various fields of medicine [46].

Patient Education Publications 

Patient education publications provide evidence-based knowledge of diseases and conditions for the affected population. While distributing credible scientific content, the language of these publications must be tailored to the comprehension of the target audience (main target audience: patients and their relatives, as well as the community). These publications do not have a large culture at present, although they are essential in preventing diseases, slowing the progression of chronic diseases and preventing the recurrence of certain acute diseases [47]. The establishment of new journals in this area is desirable and these publications should be included among the indicators on certain grant proposals. 

##### Evaluation of TM Knowledge Articles

As was mentioned at the beginning of the TM Knowledge section, many review articles and publications in general are inaccurate or biased, which is a serious obstacle for knowledge translation into healthcare benefits. Therefore, the assessment of reviews, meta-analyses or other articles by journal editors and reviewers are critically important. There are several measures that could be taken to improve the situation, including the employment of a statistician reviewer for meta-analyses and using consultative peer review. Technical innovations in the peer review system could allow corrections, retractions, automated statistical-checks and post-publication reviews [48,49]. Moreover, a good critical review should be rewarded with impact factors as well. This would increase reproducibility. Unfortunately, knowledge publications and standardisation of utilisation (review articles, guideline development and procedure of standardisation) are not rewarded according to their importance. They are not accepted as part of a PhD career in some countries, or rewarded by a high impact factor, which is an obstacle for having an impact on the health of society.

#### 3.1.4. TM Communication 

Of course, the information published in the TM Knowledge section will not be used by itself in everyday life. This information should be provided to all participants in a professionally designed manner.

##### Definition of TM Communication

**Figure 6:**
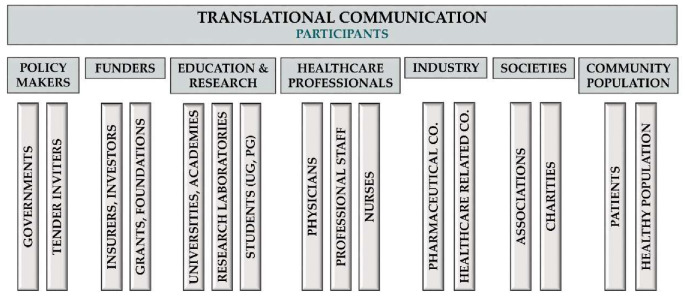
Participants of translational communication.

Translational communication is the professional dissemination of knowledge to the community, including but not restricted to patients, scientists, healthcare professionals, policymakers, insurance and pharmaceutical companies, commissioners and taxpayers. It should involve multilateral communication with a strong emphasis on receiving feedback from the participants, including patients, and reacting to it in a responsible manner (Figure 6).

##### The Importance of Bilateral Communication 

Opinion leaders, medical societies and professional organisations providing representation and advocacy in healthcare play an important role in transferring appropriate information to their members and holding courses where evidence-based clinical practice guidelines, recommendations and reports on research results are presented. In the United Kingdom, the Dissemination Centre of the National Institute for Health Research (NIHR) does outstanding work in promoting research and providing good research evidence for decision-making while reaching and educating professionals on multiple platforms. The Centre’s main strategy is to bundle evidence for target audiences and to engage these stakeholders in interpreting and making sense of the research in relation to practice. A good example of the implementation of this strategy is the successful project the NIHR ran for paramedics in 2016 on research into ambulance services. After a meeting and debate with the Ambulance Trusts, a thematic review was published that brought together and synthesised the available evidence in urgent care [50]. Creating these kinds of reviews is strongly recommended in all fields of healthcare to aid professionals and patients in making evidence-based decisions about which treatments and practices are most effective and provide the best use of resources.

##### The Importance of Responsible Communication 

Information on new scientific discoveries is usually not disseminated to the community in an effective manner, and patients’ expectations and their ability to understand the results of individual studies are not well-considered. Health literacy (the skills to access, understand, appraise and apply information to make health-related decisions) is an important determinant of health. Low health literacy is linked to adverse health outcomes and higher healthcare costs [51]. In the United States, 26% of the population has difficulty with common health-related tasks (e.g., complying with directions on medications), while the European Health Literacy Survey reports the prevalence of limited health literacy between ~ 30 and 60% among eight European countries [52]. Research results are frequently overstated by the media as ‘breakthrough discoveries’, thus worsening the competencies of the community to understand and use information in ways that supports the maintenance of good health. Scientific communities have a responsibility not to over-claim for single studies and to manage expectations of patients and the public around the timeline for appropriate clinical development and testing.

##### Channels Used for Communications 

There are many channels of communication that can be used to disseminate information in healthcare: face-to-face or personal communication, broadcast media, mobile or other electronic communication and of course the written formats. Moreover, regardless of whether we like it or not, social media has broken into every field, including medicine. It certainly has a great and growing impact on TM, as it is the easiest and most rapid way of disseminating medical knowledge and is widely used all over the world. It (1) makes it possible for relevant research findings to spread and go viral, (2) can be used in the recruitment of populations for medical research, (3) increases the chances of research being picked up by stakeholders and (4) helps to connect patients with each other (clubs) and with healthcare professionals (Question & Answer forums). However, the growing speed of disseminating scientific findings via social media can be not only beneficial, but can create a high risk of spreading fake news and generally lacks quality filtering [53]. Therefore, as noted above, regardless of the channel of communication, it is important for the information to reach the population in a peer-reviewed, planned and professional manner.

##### Benefits for Patients 

To make proper decisions on health-related questions, patients should be taught about methods of prevention and effective control, information on clinical research results, newly available therapies and lifestyle guidance (e.g., nutrition counselling and physiotherapy). Patient organisations represent one of the best channels for patient education, where members can receive reliable, professional answers to their questions, making it possible to prevent patients from receiving misleading information from unauthorised sources. Patient organisations could have a “myth-busting” function by contextualising evidence and teaching patients how to look “behind the headlines” and appropriately evaluate medical information. Continuous contact in the form of e-mails, social media, presentations and club meetings is essential to provide evidence-based information, improve compliance and offer psychological support. A description of risk factors also raises awareness and potentially reduces the recurrence of diseases. The slow integration of the latest scientific discoveries and evidence-based knowledge is also a major problem from a financial perspective. Indeed, not all countries have legal mechanisms to ensure that emerging effective treatments are translated into appropriate coverage. Nevertheless, newly available therapies should be incorporated into national insurance systems as soon as possible to maintain the best quality of healthcare. One example of bad communication between participants is the timing of a cholecystectomy after mild biliary pancreatitis. Clinical scientists reported in 2015 that the most effective and economical way is to perform the cholecystectomy during the admission for mild acute biliary pancreatitis [54]. However, almost five years after publication, in most countries, physicians still perform this procedure two–three months after an acute pancreatic inflammation. Moreover, insurance companies and healthcare providers have still not made it mandatory for institutions to perform a same-admission cholecystectomy. Notably, this practice results in an almost five-fold increase of the disease recurrence, which is unacceptable on both medical and economical grounds. This example shows that lack of effective communication between stakeholders, including decision-makers, affects implementation of clinical guidelines in everyday practice. Therefore, proactive communication is also required to target insurance companies, as the rapid integration of the latest scientific knowledge results in more effective and economical healthcare. Paying for expensive innovative therapies is a major challenge worldwide and makes the adoption of these treatments a long process, as there is increasing pressure on governments to fund more expensive therapies, policymakers need guidance to create a balance between ensuring patient access to innovation and maintaining financial sustainability [55]. 

##### Benefits for Healthcare Professionals

Of course, it is not only patients who benefit from communication. Proper, two-way communication can also benefit healthcare professionals. Communication can aid in fund raising for research and networking between clinicians, researchers and patient groups. The latter can significantly improve patients’ willingness to participate in registries or clinical trials. Fast and effective communication can accelerate the implementation of TM Knowledge into daily practice. Due to the rapid development of scientific knowledge, it is difficult even for a scientist or healthcare professional to remain familiar with new clinical nomenclature, new diagnostic techniques, new therapies and available research methodologies. Therefore, facilitating the spread of evidence based interventions in routine practice and activities in implementation science are of crucial importance [56]. 

### 3.2. Interdisciplinary Support

Many countries have realised the importance of supporting TM, established dedicated translational organisations such as the National Center for Advancing Translational Sciences (NCATS) in the US, the European Infrastructure For Translational Medicine (EATRIS), the Therapeutic Innovation Australia (TIA), the Centre for Drug Research and Development (CDRD) in Canada and MRC Technology (MCRT) in the UK, and elaborated and implemented grant programmes for this purpose [57]. Modelled on ITMAT (15), the National Institutes of Health in the United States launched the Clinical and Translational Science Award (CTSA) funding programme in 2006, with 60 projects financed between 2006 and 2011. Within these CTSA projects, special pilot funding was devoted to establishing multidisciplinary project development teams; this programme had a great influence on the development of translational research [58,59,60]. In the same period, with the world’s largest and still growing population and the problem of aging, China desperately needed solutions for its clinical needs. China has published national strategies for the development of TM, and substantial government investments have been made. As a result, 51 centres for translational research were established during this period [61,62,63]. In the UK, the NIHR established Biomedical Research Centres, analogous to the CTS institutes in the US and the Government awarded the title of Academic Health Science Centres to institutions in which CTS and the clinical mission were integrated [64]. 

Every country and institution has a different set of opportunities and challenges (existing infrastructure, education structure, grant system and community involvement); therefore, structures that support TM tend to vary greatly between countries and institutions. However, a team approach with high interdisciplinarity is necessary for delivering impact [3]. In many countries, an important hurdle for clinicians in performing investigator-initiated academic research is the lack of resources and time. Therefore, interdisciplinary research support would be useful everywhere.

#### 3.2.1. The Interdisciplinary Unit in TM 

This interdisciplinary unit may support TM Healthcare, TM Science, TM Knowledge and TM Communication, as well. This role should cover: (1) the development of translational programmes (education, training programmes and research collaborations) to facilitate communication between basic, pre-clinical and clinical research [65]; (2) the definition of key development needs to facilitate TM (e.g., efficient use of information, building an infrastructure to transform healthcare databases into a self-learning system or utilising healthcare economics tools or behavioural economics, all for the purpose of developing decision support tools); (3) the maintenance of professional knowledge of guidelines and support for research methodologies; and (4) interdisciplinary support for research (biostatistics, IT, data management and patient inclusion, ethical submissions, PR, media relations, patient club coordination, etc.) and other supporting roles (event coordination, management, administration and training in regulatory science) [66]. 

The recommended interdisciplinary support unit would consist of the following teams:

##### Medical Support Team 

The medical support team consists of coordinators whose role is to support investigators with clinical research methodology and guideline knowledge. They will guide the investigators through the process of planning, developing and launching clinical research. They connect investigators with the members of the interdisciplinary support unit. They are part of the process of realising a scientific idea, using relevant clinical investigation methodology. They carry the primary responsibility for ensuring reporting standards are met.

##### Information Technology (IT) Team

The IT team develops self-learning systems to improve access to data and to support the efficient use of data. It develops electronic research forms and validations and is responsible for anonymisation of research subjects. It also collects and provides feedback on data quality and operation of the system, thus, facilitating communication. It coordinates test procedures, operation and maintenance of the system, and error handling. Finally, the task of combining large amounts of diverse data and utilising artificial intelligence to refine and automate diagnosis and therapeutic decision-making falls within their remit.

##### Biostatistics Team

The biostatistics team supports the design of studies, their rigorous implementation and development of decision support tools (self-learning systems for healthcare databases, using big data applications), makes sample size calculations, establishes relevant statistical methodology and conducts statistical analyses. 

##### Healthcare Economics Team

The healthcare economics team facilitates the connection of healthcare economics tools to current projects. It provides economic evaluation to make the best use of clinical evidence through a systematic consideration of the effects of all the available alternatives on health, healthcare costs and other effects that are regarded as valuable [67]. It uses theoretical concepts and empirical methods in health economics to bridge the gap between the decision to fund and use a new health technology in clinical practice (the backend of TM) and the decision to invest in its development (the front end of TM) [68]. 

##### Clinical Research Administration Team

The clinical research administration team may provide valuable support in patient inclusion, data collection and upload, in-house and on-site monitoring, documentation and preparation of submissions of the registry/clinical study applications to the relevant authorities, and support for internal and external audits. The members actively participate in the development and management of clinical studies. Clinical research administrators have direct contact with patients and investigators, are part of the patient management team and provide administrative assistance in documenting clinical research. Quality monitors develop and control data collection and upload processes to ensure data quality. They carry out in-house and on-site monitoring. Monitoring reports are prepared to identify any missing or incorrect data and define steps necessary to improve data quality. The ethical regulation and documentation coordinator initiates and coordinates the authorisation of all clinical research. This person prepares and collects the required documents, submits them to the relevant authority and files all ethical documentation properly. The biobank coordinator is responsible for all duties connected to biological samples stored in the biobank. They coordinate the receipt, registration, documentation, storage and transport of biological samples. 

##### Legal Team

The legal team plays an important role in the storage of data and biobank samples. The preparation of appropriate, often multi-party contracts is no longer possible without legal assistance. The General Data Protection Regulation (GDPR) in the European Union provides protection and privacy for all individual citizens of the European Union and the European Economic Area. Legal integrity is essential for compliance with these policies.

##### Communication Team

The communication team is crucial for the proper and professional dissemination of information, as stated earlier. The team is responsible for PR, media relations and patient club coordination. It also promotes research activity, contributing to the presentation and practical use of clinical research results. It plays an important role in influencing health policy and in encouraging the healthcare system to meaningfully utilise the experiences of patients and medical personnel. One of the most important roles of this team is to maintain two-way communication with patients, provide them with information and opportunities through patient organisations, and involve them in projects in an advisory capacity. The communication team must understand the results and conclusions clearly to disseminate valid information to the target audience. 

#### 3.2.2. Quality Requirements

The quality of support depends on the expertise of the members of the interdisciplinary support unit. It is recommended that general requirements and specific education, practice and/or a proven track record of their achievements in the relevant areas be defined. However, professional education and training are missing in many countries. It is therefore crucial that programmes and courses are developed. 

In summary, the scientific idea should clearly come from the investigators. However, administrative support (registration, ethical permissions, authority audits, project management, etc.), specific knowledge of methodologies and direct contact and communication with investigators, patients, healthcare providers and other members of the community may accelerate the process of TM. We believe that a good academic medical centre should provide the core facility for interdisciplinary research support.

### 3.3. Academic Aspects

#### 3.3.1. Undergraduate Education

As noted in the introduction, understanding the methodologies that make new discoveries is essential for the proper use of TM Knowledge. This is particularly important in clinical research, as the results obtained here can immediately be used for the benefit of the population. Clinical scientific methodologies have largely evolved over the past two decades, whereas many medical universities change their curriculum too slowly and are not prepared to teach clinical methodologies and TM. Therefore, medical schools are strongly advised to plan compulsory or at least elective courses for medical students to teach them the basics of TM, scientific methodologies and scientific knowledge supplemented by techniques of effective translation of medical information for the different members of the community. 

Although the role of nurses is also critical to the research enterprise, clinical research nursing is not included in nursing curricula. In addition to providing clinical care, nurses taking part in clinical research play a role in the maintenance of participant safety, the integrity of protocol implementation and the accuracy of data collection [69]. Regarding the required expertise, nurses should also receive education on the actual process of clinical and translational research and regulatory requirements in the form of a master’s programme or a specialised course. 

#### 3.3.2. Postgraduate Education

Integrating TM into postgraduate education may seem easier, as it is less demanding than undergraduate university education. However, a significant number of supervisors may have little or no knowledge about new clinical methodologies. Furthermore, in the absence of interdisciplinary units, clinical projects can be extremely slow to complete, and they are therefore not very popular among students. The University of Pecs TM Centre has developed a new form of postgraduate education that includes continuous three-way communication between supervisors, methodologists and postgraduate students, which can allow training of larger numbers of students (https://tm-centre.org/hu/tm-projects-1/). This ‘learning by doing’ model can provide students with effective training within 12 to 24 months. It is important that policies and regulations for PhDs and other postdoctoral qualifications not only allow new original publications leading to a degree, but also secure appropriate quality of all TM areas for granting such degrees. Not only can this form of education provide research opportunities for those seeking a PhD, but its abbreviated form may allow graduated physicians or residents to acquire proficiency in TM, which is essential for making thoughtful decisions in practice. In order to translate scientific results into everyday practice, it is essential to train those involved in patient care. Governments and healthcare providers should therefore develop a feasible methodology for training practicing clinicians.

## 4. Discussion and Future Perspectives

In summary, science has come to the forefront of healthcare. Translational thinking is essential for translating and implementing scientific results into everyday practice. However, this is a far cry from the current state of affairs in the European Union. The European Statistical Office of the European Commission has recently reported that 1.7 million people under 75 years of age died in Europe in 2016, and around 1.2 million of those deaths could have been avoided through effective public health and primary prevention. A detailed analysis showed that around 80% of deaths in Eastern Europe and still around 60% in Western Europe were either preventable or treatable. Therefore, there is clearly an unmet need to understand the challenges and difficulties of 21st century healthcare (Table 1). 

TM is certainly one of the enterprises which, if expanded more broadly, could save lives and elevate the health level of the population. However, as discussed in this article, we need a broader interpretation of TM. It is difficult to predict healthcare for the future, but we can be certain that understanding science and utilising scientific knowledge will be even more important than it is today. Evidence-based medicine has been demonstrated to be better than previous individual experience-based medicine, but there are indicators that personalised therapies can provide even more efficient and cost-effective healthcare. Artificial intelligence, genetically-based patient care or drug testing on organoids grown from patient cells are likely to be an integral part of healthcare in the future. Consequently, healthcare strategies used by universities, hospitals and academies must be subject to continuous review and renewal.

The authors of this paper, including several senior officers of Academia Europaea, hope to prompt, with this work, discussion of new paradigms of the European healthcare system.

## Figures and Tables

**Table 1:** Identified problems and suggested solutions.

TM HEALTHCARE	**IDENTIFIED PROBLEMS**	
	1	Lack of hospital-level quality assessment and feedback makes it impossible to define where the best quality treatment is available.
	2	Although we know that the multidisciplinary approach is essential to ensure good quality and effective patient care, it is still not applied in many cases.
	3	Only a narrow area of patient care is involved in scientific activities or clinical research.
	4	Funding is not sufficient for covering the costs of scientific activities (staffing, consumables, facility development) in many healthcare centers.
	**SUGGESTED SOLUTIONS**	
	1	The widespread adoption of science in patient-care is needed for improving the quality and cost-effectiveness of healthcare.
	2	Multidisciplinary teams should be formed everywhere to increase safety and achieve efficiency and added value in patient care.
	3	Continuous data recording and monitoring are needed for hospital-level assessment of quality and effectiveness to define the necessary changes in the structure of healthcare (treatment centers) or funding.
	4	Biological sample collection and cooperation of healthcare centers and research centers are necessary to promote scientific activity.
	5	Tertiary centers may be ideal locations to start implementing the TM Healthcare system
	6	All healthcare providers and other disciplines should agree on evidence (science)-based patient management and work together in a multidisciplinary approach.
	7	General patient care should be financed by national and private health insurance. Activities intended to achieve new scientific results should be funded by institutional, national and international grants and funds. Current budgets should be elevated as well.
TM SCIENCE	**IDENTIFIED PROBLEMS**	
	1	Many discoveries do not reach patient care.
	2	Clinically relevant questions are not addressed through the arsenal of basic research methodologies.
	3	The regional differences in funding and opportunities. (In Western Europe, funding of basic science is inadequate in relation to the scientific opportunities that exist. Whereas clinical science is less developed in Eastern Europe).
	**SUGGESTED SOLUTIONS**	
	1	Close cooperation of healthcare centers and research centers are necessary to promote scientific activity.
	2	All publications describing new, previously unknown results should be considered as original publications, regardless of whether these analyses are based on newly generated or already existing data
	3	Funding has to be tailored to research needs. A special emphasis should be put on raising budgets for science in Eastern and Central European countries.
TM KNOWLEDGE	**IDENTIFIED PROBLEMS**	
	1	Readers, professionals cannot cope with the huge number of publications, knowledge released.
	2	Many review articles are inaccurate or biased.
	3	There is a huge gap between the guideline, knowledge and its implementation.
	4	Summary publication and standardization is not rewarded in the academic carrier, neither in impact factors.
	**SUGGESTED SOLUTIONS**	
	1	A proper classification of summary publication is needed, our paper recommends one.
	2	Quality assessment of summary publications is extremely important. Several measure are recommended in the peer review process (consultative peer review, technical innovations, etc.)
	3	A good critical review should be rewarded with impact factors as well.
	4	Summary publication, standardization is an important part of the TM cycle, the field should be rewarded in academic progress and in publication impact.
TM COMMUNICATION	**IDENTIFIED PROBLEMS**	
	1	Often there is a lack of communication between the participants of healthcare (e.g., scientists and insurance policy-makers or scientists and patients).
	2	Guidelines are often not translated into the local language and not incorporated into insurance policies in many countries, which is an obstacle for implementation.
	3	Patient organizations and advocacy is underdeveloped in many countries.
	4	Medical students and nurses has no access to clinical research methodology knowledge as the curricula do not cover them.
	**SUGGESTED SOLUTIONS**	
	1	Bilateral/multilateral communication need to be developed between participants of healthcare. Feedback has a crucial importance.
	2	Guidelines need to be translated and incorporated into insurance policies, knowledge publication should be communicated to healthcare professionals in order to be implemented.
	3	Patient organizations have a critical role as channels for patient educations and advocacy, in most countries patient organizations have to be developed.
	4	All research methodologies (including clinical research) should be included in the curricula of medical universities and education of nurses.
	5	Policymakers need guidance to create a balance between ensuring patient access to innovation and maintaining financial sustainability
TM INTERDISCIPLINARY	**IDENTIFIED PROBLEMS**	
	1	Lack of time, resources for organizing clinical research among clinicians.
	2	Lack of clinical research methodology knowledge and special knowledge in the fields of biostatistics, IT, communication to policymakers, etc. among clinicians and researchers.
	3	A dedicated interdisciplinary team for supporting the TM cycle elements is missing from the academic organization in many countries, especially in Central and Eastern Europe.
	**SUGGESTED SOLUTIONS**	
	1	Providing funds for and establishing interdisciplinary teams in the academic environment supporting TM.
	2	The interdisciplinary team should cover the fields of biostatistics, IT, data management and patient inclusion coordination, ethical submissions, communication, patient club coordination, implementation coordination, other supporting roles like event coordination, management, administration and training in regulatory science, etc.
TM ACADEMY	**IDENTIFIED PROBLEMS**	
	1	Universities and academic institutes do not adapt fast enough to the changing environment, concerning the inclusion of new methodologies in their curricula.
	2	Clinical research nursing is not included in nursing curricula, although they play a critical role in play a role in the maintenance of participant safety, the integrity of protocol implementation and the accuracy of data collection.
	3	Because of the lack of knowledge in clinical research methodologies among supervisors and interdisciplinary support units, clinical research is not very popular among students.
	**SUGGESTED SOLUTIONS**	
	1	Medical schools are strongly advised to plan compulsory or at least elective courses for medical students to teach them the basics of TM, scientific methodologies and scientific knowledge supplemented by techniques of effective medical information translation for the different members of the community.
	2	Nursing curricula should include clinical research nursing.
	3	New form of education is needed, a ‘learning by doing’ model, which may involve practicing physicians beside student and those seeking PhD.

## Supplementary Materials

The following are available online at https://www.mdpi.com/2077-0383/9/5/1532/s1, Table S1: Definitions.
