# Supplementary material for: Academia Europaea Position Paper on Translational Medicine: The Cycle Model for Translating Scientific Results into Community Benefits
Source: J Clin Med. 2020 May 19;9(5):1532. doi: 10.3390/jcm9051532 (PMC7290380; doi:10.3390/jcm9051532)
Supplement: Supplementary file 1 [file jcm-09-01532-s001.pdf]

	DEFINITIONS
TM	TM is an enterprise which turn science for healthcare benefits.TM focuses equally on healthcare, the acquisition of new scientific results, the comprehensible and digestible summarization of results, and their communication to all participants.
TM CYCLE	TM Cycle represents the methodology, how science can be utilized for healthcare benefits. Questions should arise in Healthcare (TM Healthcare) and answered through scientific activity (TM Science). New results should be summarized (TM Knowledge) and communicated to all target population (TM Communication).
TM HEALTHCARE	TM Healthcare is the starting and finishing point in translational medicine. TM Healthcare means high-quality patient care, driven by and contributing to high-quality clinical research. It can significantly boost the efficiency of clinical research, and it promotes efficient communication and cooperation between all stakeholders.
TM SCIENCE	TM Science is defined as any act that entails new discoveries or observations, which help to form our knowledge of the human body and its interactions with the environment and carry a clear hope of attaining novel achievements for the benefit of human health (regardless of whether these new discoveries are based on analyses of newly generated or already existing data). TM Science cover basic, applied and clinical sciences.
TM KNOWLEDGE	TM Knowledge aims to summarize the discoveries of TM Science to facilitate the translation of scientific findings to community benefits. Its language and the way of writing should be chosen in a way that is understandable to the target audience.
TM COMMUNICATION	TM Communication is the professional dissemination of knowledge to the community (patients, healthy population, scientists, healthcare professionals, policymakers, insurance and other companies, commissioners and taxpayers). It should involve multilateral communication with a strong emphasis on receiving feedback from the participants including patients and reacting to it in a responsible manner.
